# Dr. Gustavus Pierce English

**Published:** 1917-06

**Authors:** 


**Dr. Gustavus Pierce English,** L. I. C. H. 1882, died at his home in Utica Feb. 15, aged 59.

